# Impact of cut-point methods on classification of physical activity and sedentary behaviour of toddlers

**DOI:** 10.1186/s12889-025-24636-6

**Published:** 2025-10-02

**Authors:** Jill Marie Ferry, Joaquin Escribano, Mariona Gispert-LLauradó, Berthold Koletzko, Veit Grote

**Affiliations:** 1 Department of Paediatrics, Dr. von Hauner Children’s Hospital, LMU University Hospital, Munich, Germany; 2 German Center for Child and Adolescent Health, site Munich, Munich, Germany; 3 Department of Paediatrics, Nutrition and Development Research Unit, Universitat Rovira i Virgili, IISPV, Reus, Spain; 4 Department of Paediatric, Hospital Universitari Sant Joan de Reus , Reus, Spain

**Keywords:** Accelerometer, Toddlers, Cut-points, Physical activity, ActiGraph, Sedentary behaviour

## Abstract

**Background:**

Classification of physical activity (PA) depends on the cut-point method used to allocate PA counts from accelerometer measurements. This study investigates how three validated cut-point methods affect the time spent in various levels of PA and sedentary behaviour (SB), and how they impact toddlers estimated adherence to PA guidelines.

**Methods:**

PA was assessed using an ActiGraph wGT3X-BT accelerometer in a cohort of 653 two-year-old children participating in the Toddler Milk Intervention study. Children wearing the ActiGraph for at least four days, with a minimum of six hours wear-time per day, were included. Time spent in SB and different activity levels were estimated according to three cut-point methods and were standardized to individual mean wear-time. We used one cut-point method based on the vertical axis (VA) (Trost VA), with an epoch length of 15 s and two cut-point methods based on either the VA (Costa VA) or on the vector magnitude (VM) (Costa VM) with an epoch length of five seconds. Estimates of SB and PA for each method were compared with repeated measures ANOVA.

**Results:**

The time toddlers spent in PA was significantly different depending on the cut-point methods. Costa VM classified on average 62 min (95% CI 61, 64] more per day as SB and 57 min (95% CI -58, -56] less per day as LPA compared to Trost VA (both *p* < 0.0001). For MVPA, the mean difference between Costa VA and Trost VA was 6.8 min (95% CI -7, -6; *p* < 0.0001). Concurrently, the proportion of children meeting the WHO recommendation of 180 min of total PA differed between cut-point methods, with 86% according to Costa VM and 97% according to Trost VA.

**Conclusions:**

The time toddlers engage in different intensities of PA is significantly determined by the selection of cut-point method. Notably, the use of a different cut-point method leads up to a 10% difference in the estimated time spent in LPA and SB, but only a 1% difference of moderate-vigorous PA. These differences change the estimated adherence to recommendations. Future research is needed to standardize the data processing methods for better comparability between studies analysing toddlers’ PA.

**Registry:**

ClinicalTrials.gov, TRN: NCT02907502, Registration Date: 31 August 2016

**Supplementary Information:**

The online version contains supplementary material available at 10.1186/s12889-025-24636-6.

## Background

Physical activity (PA) is essential for the healthy development of toddlers, providing numerous benefits that extend well beyond physical health e.g. cognitive and motor skill development [1]. In early childhood, PA contributes to favourable health outcomes, including cardiometabolic health, psychosocial well-being, and skeletal health [1, 2]. In general, a greater degree of PA in early childhood is associated with greater health benefits [2]. On the contrary, prolonged times of sedentary behaviour (SB) during early childhood are associated with untoward health outcomes such as adiposity and negative psychosocial health outcomes [3]. For toddlers 1–2 years of age, the World Health Organization (WHO) recommends at least 180 min of PA per day at any intensity, while keeping sedentary screen time or uninterrupted sitting below one hour at a time [4]. The majority of toddlers accumulate 180 min of TPA per day [5], whereas large proportions of European children from 2 to 9 years do not reach the recommended levels of PA [6].

During toddlerhood, from 1 to 3 years of age, PA involves unstructured play and structured activities like active play programs to improve toddlers´ motor skills [7]. To enhance PA in toddlers, it is essential to understand why, when, and how much PA they engage in. Toddler PA can be quantified with accelerometers in three-dimensional accelerations. The three dimensions are the vertical (Y), the horizontal right-left (X) and the horizontal front-back axis (Z). In this age group, accelerometers are typically worn at the hip [8], while wrist or thigh placements need different calibration and data processing methods [9]. Due to toddler specific behaviours, a wrist placement in toddlers can produce more noise compared to a hip-worn monitor [10], whereas a thigh placement can be used only for some devices and is impractical for others due to size [11]. Various solutions exist for post-processing accelerometer data, including both commercial software such as *ActiLife* [12] and open-source packages like *GGIR* (R package) [13]. The raw accelerations can be used to quantify PA and SB through metrics such as the Euclidian norm of raw accelerations – 1 g (ENMO) [14, 15], whereas the data can also be processed to the arbitrary unit of activity counts [16]. The counts obtained in a given period are linearly related to the intensity of the subjects´ PA during this period [16].

Processed acceleration data, such as activity counts, are classified into moderate PA (MPA), vigorous PA (VPA), light PA (LPA), and SB using cut-point methods developed for specific age groups. As toddlers engage in brief periods of MPA and VPA [5], these intensities are usually aggregated into a unified moderate to vigorous PA (MVPA) category.

Validated cut-point methods correspond to specific age groups using a fixed combination of measurement axes and epochs choice [17, 18]. The cut-points are usually developed in calibration studies with a small sample size, where thresholds of PA levels and SB are defined by direct child observation and classified with the Children’s Activity Rating Scale (CARS) [17–19]. Cut-point calibration studies, by design, capture only a subset of activities, which may not fully represent the diverse range of movements encountered in daily life [17, 18]. There are cut-points based on data of three-dimensional vector magnitude (VM) or on the vertical axis (VA). Cut-points based on the VA have been derived from older studies using uniaxial accelerometers. The vector magnitude (VM) is the square root of the sum of squared activity counts of all three axes:

$$\mathrm{VM}=\:\sqrt{x^2+y^2+z^2}$$

Incorporating information from all three axes is especially beneficial if the monitor did not remain correctly positioned. Consequently, the VM is less impacted by an inaccurate accelerometer alignment [20]. For young children, short epoch lengths, from 1 to 15 s are recommended to capture short activity bursts [21–23]. Different activity intensities are averaged within an epoch length [24]. Research with pre-schoolers indicates that using different cut-point methods on the same dataset can lead to significant statistical differences in estimated PA levels [25–29]. In a toddler validation study, cut-points originally developed for pre-schoolers demonstrated poor to fair overall accuracy when compared with direct observation [30]. Additionally, the application of cut-points is in some studies not consistent with the settings of the calibration study [25, 29]. For instance, the use of a different epoch length than in the calibration study can lead to a significant change in PA and SB estimates and thus creates misleading results [23, 26].

Thus, differences in analysis methods used for accelerometer data from toddlers can result in varying levels of PA and SB. The main objective of the present study is to characterize the impact of the application of three different cut-point methods from calibration studies on the estimated time spent in various levels of PA and SB in a large toddler study population. Furthermore, the study evaluates how the cut-point methods affect the adherence to WHO guidelines.

## Methods

### Study subjects and procedure

**Fig. 1:**
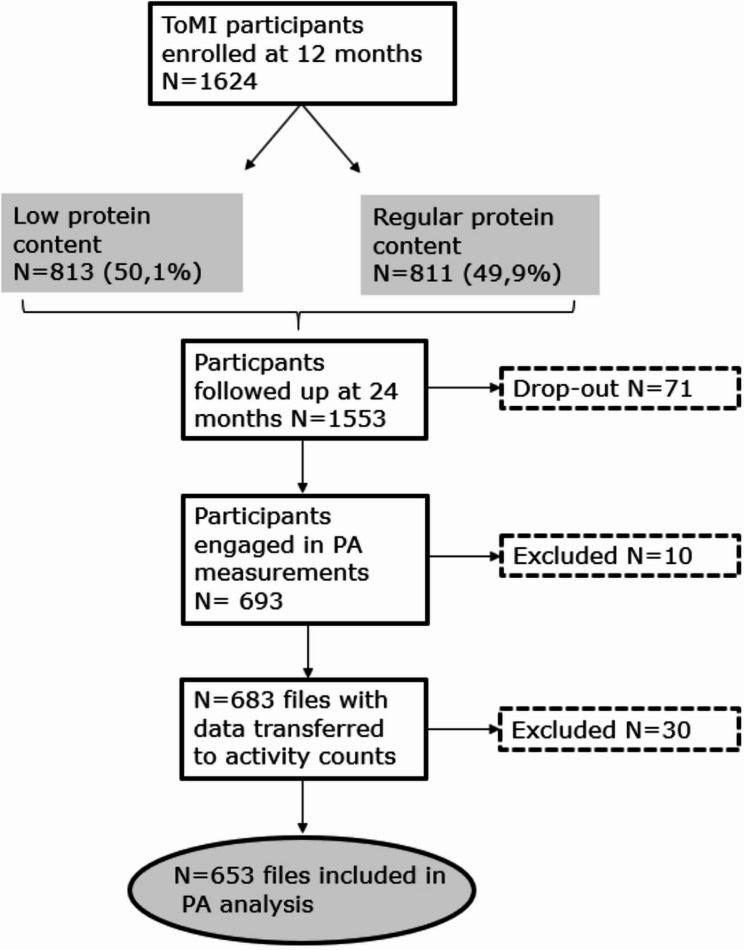
Study population ToMI physical activity participants at 24 months of age

The study population is a subsample of the Toddler Milk Intervention (ToMI) trial (Trial Registration NCT02907502, https://clinicaltrials.gov/). The double-blind, randomized, longitudinal intervention.

study recruited healthy infants aged about one year of age (between 11.5 and 13.5 months) from 2016 onwards in Munich (Germany) and Tarragona/Reus (Spain) [31]. The main goal of the study was to investigate how toddler milk with different protein content during the second year of life influences weight development and the risk of overweight in healthy children [31]. 1624 children have been enrolled in the ToMI study (see Fig. 1). At the 2-year follow-up visit, PA was assessed in a subsample of children (*n* = 693) using the ActiGraph wGT3X-BT accelerometer (ActiGraph LLC, Pensacola FL, USA). This subsample included children whose parents consented to PA measurements. Based on the results of Bisson et al. (2018) toddlers wearing the ActiGraph for at least four days with a minimum of six hours of wear time per day were included in the data analysis [32]. Activity files which failed inclusion requirements or corrupted data in the raw files were excluded from the analysis.

The ActiGraph was initialized with the idle sleep mode enabled and a sampling rate of 30 Hz, because human movement is usually observed below 10 Hz, thus according to the Nyquist Theorem [33] a sample rate of 30 Hz is considered sufficient to capture children´s PA [34]. The accelerometer was handed out to the parents during the study visits and the correct application was explained. PA monitors were attached to the right hip and oriented according to the three axes. Participants were asked to wear the device for 7 days and remove the device during bedtime, naps and water-based activities. A wear-time protocol was completed by the parents (Supplementary material file A).

### Accelerometer data processing

Data processing of raw.gt3x files was done in R (Version 4.2.2). Raw files were automatically processed into R and zeros were imputed for the idle sleep mode with the “read.gt3x” R-package [35]. Subsequently, acceleration data was calculated into activity counts per second using the “agcounts” R-package [16]. In addition, the wear time was truncated to the start and end date and time (+/- 1 h) of the wear time protocol. Removal of non-wear time was performed using Choi’s algorithm with 20 min of consecutive zeros on the VM and a 2-minute interval of non-zero counts for artefactual motion detection [36]. Based on Esliger et al. (2005), a maximum of 17.5 min of motionless data is biologically plausible in children, implying that 20 consecutive minutes of zeros can effectively identify non-wear time [37]. For PA and SB categorization the Trost et al. (2012) VA cut-points for toddlers were applied, as they are widely referenced in the literature for this age group [5, 23]. For comparison PA and SB were also analysed using the Costa et al. (2014) cut-points based on VA and on the VM [17]. The epoch length was adapted to the epoch length of the cut-point´s calibration study, which was 15 s according to Trost et al. (2012), and 5 s according to Costa et al. (2014) as outlined in Table 1.

**Table 1:** Validation studies, measurement axis, age group and epoch length used for cut-points of activity counts

Validation study	Type	Age group	Epoch	SB	LPA	MVPA
(Trost et al., 2012)[18]	VA (Y-axis)	2 years	15 s	0 ≤ 25	26–419	≥ 420
Costa et al., 2014) [17]	VA (Y-axis)	2–3 years	5 s	0 ≤ 5	6–164	≥ 165
Costa et al., 2014) [17]	VM	2–3 years	5 s	0 ≤ 96	97–361	≥ 362

*MVPA* moderate-vigorous physical activity, *LPA* light physical activity, *SB* sedentary behaviour, *VM* vector magnitude, *VA* vertical axis

Average time spent in SB and PA levels and wear time were calculated over all measurement days. In addition, these average times in the different activity levels were adjusted for possible differences in wear time, which may affect the minutes in PA or SB, as applied in the IDEFICS study [38]. For this purpose, the minutes spent in each activity level were divided by the participant’s wear time and then multiplied by the average wear time of all children.

### Statistical analyses

Statistical analyses were performed in R (Version 4.2.2). Descriptive data are reported as means and standard deviation (SD) for continuous variables (e.g. BMI and age). Nominal variables such as sex, season and country and categorical variables (e.g. maternal education) are reported as number (n) and percentage (%). For group comparisons paired t-tests were performed.

A repeated-measures analysis of variance (ANOVA) was conducted to examine differences in wear-time-adjusted PA and SB minutes across the three cut-point methods. The repeated-measures ANOVA included the within-subject factor “cut point method” and subject ID as a random effect. Post-hoc analyses were performed using estimated marginal means with Bonferroni correction to identify pairwise differences. Statistical significance was assumed at a maximum error probability of 0.05.

Z-scores of anthropometric measurements were built based on the WHO Growth Reference Study with normal weight BMI-z <= + 1SD and overweight/obese BMI-z > + 1 SD [39, 40].

## Results

### Study population

Six-hundred ninety-three participants completed PA monitoring with the ActiGraph (Fig. 1). Activity counts could be produced from 683 participant files, and 653 participants met the wear time criteria. The ActiGraph was worn on average on 6.6 (SD ± 0.8) days with an average wear time per day of 10.4 (SD ± 1.3) hours.

Mean age was 24.1 months (SD ± 0.6) (Table 2). 55% of the participants were male and 56% were living in Germany. 21% of the study population were overweight or obese with a mean BMI z-score of 0.3 (SD ± 0.9). No significant differences in PA or SB were observed between the intervention and control group. 53% of mothers reported a high education status, whereas only 7% had a low education status.

**Table 2:** Study population characteristics

Characteristics		*N* = 653
Age (in months)		24.1 (SD ± 0.6)
Sex	Female Male	293 (45%) 360 (55%)
BMI	kg/m² Z-score	16.2 (SD ± 1.2) 0.3 (SD ± 0.9)
Country	Germany Spain	365 (56%) 288 (44%)
Maternal education status^1^	Low Middle High	41 (7%) 263 (40%) 349 (53%)
Season of measurement^2^	Spring Summer Autumn Winter	158 (24%) 183 (28%) 145 (22%) 167 (26%)

^1^Maternal education status categorized based on ISCED (International Standard Classification of Education 2011) levels: low 0–2; Middle 3–5; High 6–8

^2^ Seasons are categorized based on the start date of the measurement: Spring (March, April, May), Summer (June, July, August), Autumn (September, October, November), and Winter (December, January, February)

### Comparison of cut-points

The application of the three different cut-points resulted in MVPA ranging from 50 to 57 min per day and LPA varying from 193 to 251 min per day (Table 3), resulting in a TPA between 245 and 308 min per day. SB ranged from 320 to 382 min per day with the highest difference between the Costa VM cut-point and the Trost VA cut-point.

**Table 3:** Minutes (SD) spent in different PA levels and SB per day according to different cut-points^1^

	Costa VM	Costa VA	Trost VA
MVPA	52 (16)	50 (15)	57 (20)
LPA	193 (26)	215 (26)	251 (29)
TPA	245 (36)	265 (36)	308 (40)
SB	382 (36)	362 (36)	320 (40)

*MVPA* moderate-vigorous physical activity, *LPA* light physical activity, *TPA* total physical activity, *SB* sedentary behaviour

^1^Minutes adjusted to wear-time according to the IDEFICS study [38]

**Fig. 2:**
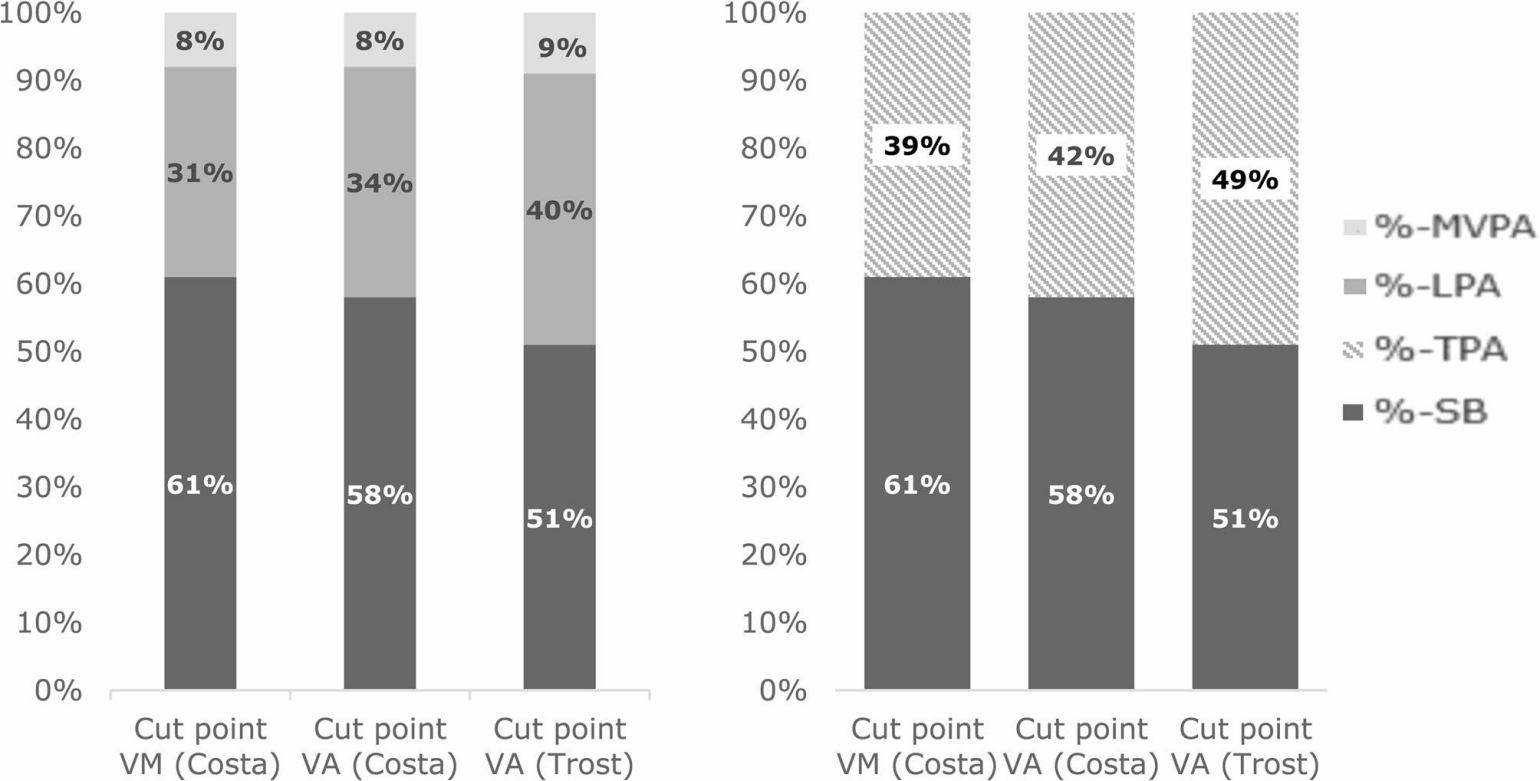
Proportion of time in different activity levels at 2 years and choice of cut-point

With the application of the Trost VA cut-point toddlers spent similar amounts of time in SB and TPA, with only 12min per day more SB compared to TPA. However, the Costa VM and VA cut-point resulted in more SB compared to TPA, 137min and 97 min, respectively. Figure 2 shows the proportion of time toddlers spent in TPA, which was significantly different depending on the choice of cut-points.

Specifically, Costa VM lead to significantly higher SB and lower TPA compared to Trost VA. Similarly, LPA and MVPA differed between cut-points, with Trost VA resulting in more PA and less SB than both Costa cut-point methods. The comparisons of cut-point methods across PA and SB were statistically significant (*p* < 0.001), as reported in Table 4. Costa VM resulted in significantly lower LPA estimates compared to Trost VA, with a mean difference of 57min per day (95% CI: [−58.26, −55.88], *p* < 0.0001). For MVPA, Costa VA resulted in 7min less per day compared to Trost VA (95% CI: [−7.44, −6.14], *p* < 0.0001). SB differed by an estimated mean difference of 62 min more for Costa VM versus Trost VA (95% CI: [61.11, 63.76] *p* < 0.001).

**Table 4:** Pairwise comparisons of cut-points across PA levels and SB

PA level	Comparison	Estimate	SE	t-value	*p*-value	CI-interval
*SB*	CostaVA – CostaVM	−20.1	0.6	−36.3	< 0.0001	[−21.37, −18.72]
	CostaVA – TrostVA	42.4	0.6	76.7	< 0.0001	[41.06, 43.71]
	CostaVM – TrostVA	62.4	0.6	113.0	< 0.0001	[61.11, 63.76]
MVPA	CostaVA – CostaVM	−1.4	0.3	−5.2	< 0.0001	[−2.05, −0.75]
	CostaVA – TrostVA	−6.8	0.3	−25.1	< 0.0001	[−7.44, −6.14]
	CostaVM – TrostVA	−5.4	0.3	−19.9	< 0.0001	[−6.04, −4.74]
LPA	CostaVA - CostaVM	21.45	0.5	43.3	< 0.0001	[20.27, 22.64]
	CostaVA – TrostVA	−35.61	0.5	−71.8	< 0.0001	[−36.79, −34.42]
	CostaVM – TrostVA	−57.06	0.5	−115.1	< 0.0001	[−58.26, −55.88]

Estimates reflect mean differences in adjusted minutes, *SE= *standard error

t-value from repeated-measures ANOVA

p-value adjusted via Bonferroni

CI-interval = 95% Bonferroni-corrected confidence interval

*MVPA* moderate-vigorous physical activity, *LPA* light physical activity, *SB* sedentary behaviour

**Fig. 3:**
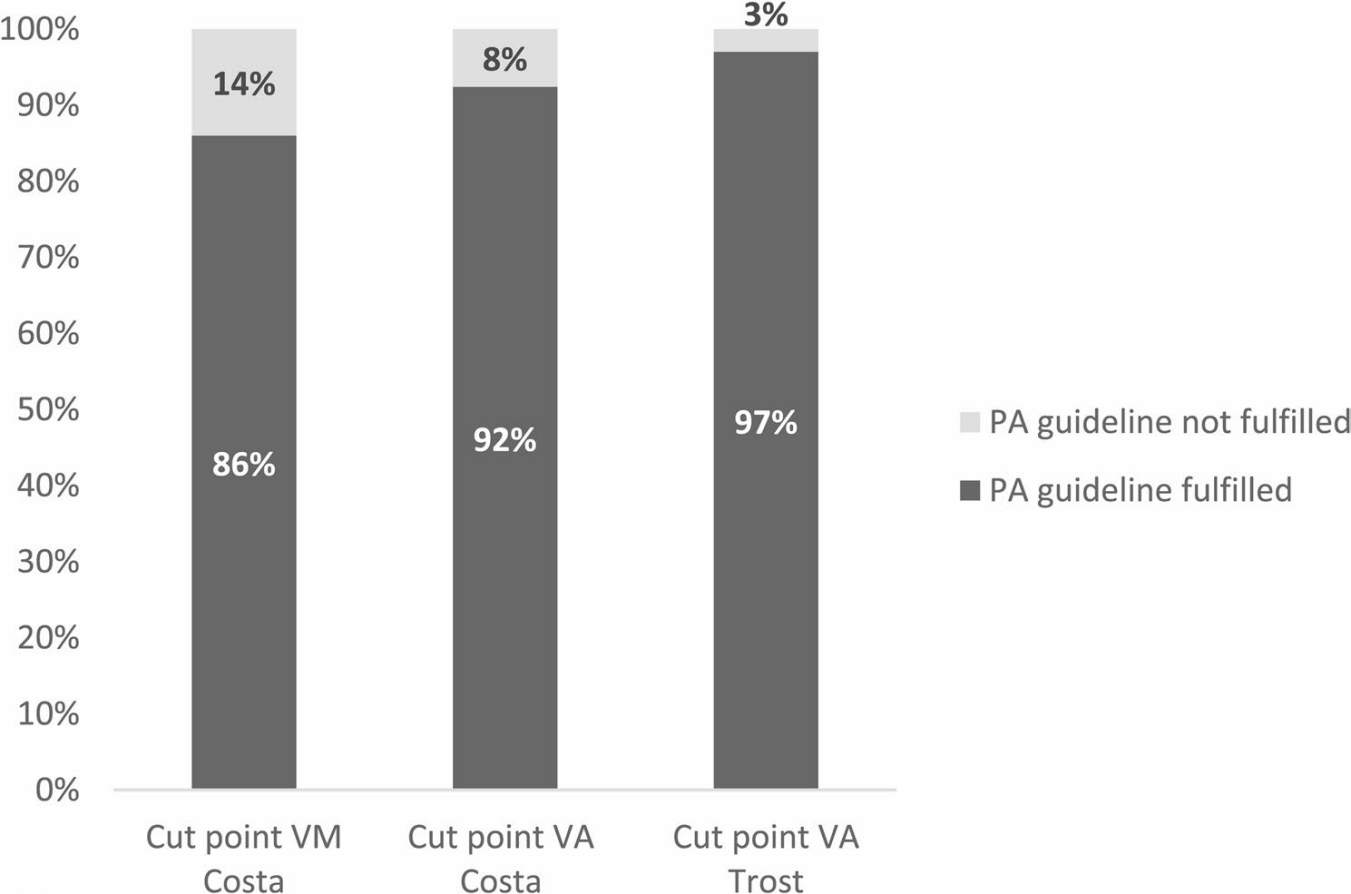
Percentages of children fulfilling WHO-guideline on PA applying different cut-point methods

Concurrently, the adherence to WHO PA guidelines differed depending on whether Costa VM or Trost VA cut-points were applied; accordingly, either 86% or 97% (*p* < 0.001) of children met the recommendation, respectively (Fig. 3). The Costa VA cut-point fell in the middle, with 92% of children meeting the recommendation. Nearly all participants (97–99%) fulfilled the recommendation of limiting SB to under one hour at a time.

## Discussion

This study aimed to compare three published cut-point methods to classify accelerometer data into PA levels and SB in a healthy, free-living toddler population and determined adherence to WHO PA guidelines. Results revealed significant differences in time spent in activity levels and SB based on the three cut-points methods applied. These discrepancies led to different categorizations regarding WHO guideline adherence. Using the Trost VA cut-point, 97% of participants met PA recommendations. In contrast, the Costa VM and VA cut-point showed a lower PA guideline adherence with 86–92%.

Based on our findings, toddlers engaged in TPA for approximately 245 to 308 min per day. This is consistent with a meta-analysis which reported an average TPA of 246 min per day (95% CI: [190, 302]) for toddlers [5]. Regarding SB, our results indicated a range of 320 to 382 min per day, in line with the meta-analysis average of 337 min per day (95% CI: [273, 401]). The findings suggest that most toddlers exceed the WHO recommendation of 180 min of TPA per day. However, most studies, including ours with 50–57 min per day, report that MVPA is on average less than 60 min per day [5]. As a result, the majority of the 180 min per day consist of LPA. MVPA has been seen to be associated with greater health benefits compared to LPA in preschool children [2, 41].

There is considerable variation in the time toddlers spend in different activity levels across studies [5, 42]. Measurement settings, such as sample frequency, device placement, and device characteristics, along with post-processing decisions like cut-point values, axes, epoch length and non-wear algorithms can influence results [26]. The lack of standardization in data collection methods and accelerometer data processing criteria creates difficulties in comparing estimates of toddlers’ PA and SB across various studies [23, 43]. It is recommended to apply the same criteria as in the cut-point method calibration study [23]. Consequently, we analysed our data using the same cut-point value, epoch length, age group and axes as in the calibration study.

The acceleration axis and epoch length employed with the cut-point value can significantly influence the assessment of PA and SB time. Combining three axes in the VM improves agreement between accelerometer-derived PA estimates and reference methods, such as direct observation. In addition, VM cut-points classify more time as MVPA than VA cut-points [44, 45]. Our results showed the Costa VM cut-point estimated 2min more MVPA time compared to the Costa VA cut-point, probably because it includes movement on other axes. VM cut-points require either substantial motion in one axis or minor motions across several axes within an epoch to label it as PA. This sensitivity enables the recognition of slight movements common in SB, and thus facilitating a clear differentiation between SB and PA [46].

Epoch length can significantly impact the time spent in various PA levels and SB, as demonstrated in previous studies [24, 47, 48]. Longer epoch lengths increase the likelihood of classifying SB as LPA because they reduce the chance of capturing variations in PA intensity [9, 47]. Longer epoch lengths average MVPA and SB into the medium intensity level LPA [24, 48]. Consistent with this observation, our analysis revealed that cut-point methods with longer epoch lengths resulted in reduced time in SB.

Also, the selection of non-wear time criteria significantly impacts the measured PA and SB and there is to our knowledge no validated algorithm for toddlers [49, 50]. The study employed the Choi algorithm [36] with a 20-minute non-wear epoch, while a comparable study [25] used the Choi algorithm with a 60-minute non-wear interval. This methodological difference is important to consider, as the Choice of non-wear time criterion can affect PA estimates by up to 10% and SB by up to 17% respectively [49]. Longer non-wear criteria risk misclassifying non-wear time as SB or LPA, while shorter criteria may cause the opposite misclassification [26, 49]. Applying the Choi algorithm with a 60-minute threshold showed a strong agreement with the logbook in toddlers, but algorithms with shorter intervals showed lower mean absolute differences [50]. To account for differences in wear time, all PA estimates in our study were adjusted for average wear time [38]. Furthermore, the parents were asked to take off the device during naps to prevent nap periods from being incorrectly classified as SB [50].

Another factor to consider is the age group. During the 1–3 years age range of toddlerhood, motor skills advance quickly, thus even minor age differences may meaningfully impact movement patterns. For instance a validation study with 17-month-old Malawian toddlers, reported notably lower cut-point thresholds on the VA and VM while using a 15 s epoch length compared to those applied in our study [51]. The Costa VA and VM cut-point methods were derived from a study population of toddlers aged 2.86 ± 0.60 years and validated with toddlers aged 2.99 ± 0.48 years [17]. Consequently, their study population was slightly older than ours. According to previous studies with toddlers, a higher age significantly increased PA in higher intensities [52, 53]. A lower vertical acceleration can be attributed to a smaller body size [10]. Regarding the Trost VA cut-point, the age of their study population aligns closely with our participants, having a mean age of 2.1 ± 0.4 years [18]. An additional age-specific challenge in assessing toddlers’ PA and SB is the identification of external motion (e.g., being carried or pushed in a stroller), which is often neglected when applying cut-point methods [5, 10]. Resolving this challenge may involve the implementation of machine-learning approaches which demonstrated an 89% accuracy in distinguishing being carried from running, walking, crawling, and climbing in one study [10]. Cut-point-based classification likely results in the misclassification of external motion in toddlers as MVPA, instead of SB, due to the relatively high activity counts. This misclassification can additionally lead to a misinterpretation of TPA and SB of toddlers [10].

Furthermore, the classification of PA and SB in toddlers is influenced by the variability of methods applied in cut-point calibration studies [44]. For instance, while both the Costa and Trost cut-point methods were validated using the Children’s Activity Rating Scale (CARS) [19], they applied different classification thresholds for the observation of activity levels [17, 18]. Both calibration studies were conducted in childcare settings with small sample sizes (18 and 22 toddlers, predominantly female), which may not adequately capture the full range of daily activities required for free-living PA assessments [44].

The collective impact of the aforementioned factors is referred to as the “cut-point conundrum,” underscoring the complexities involved in selecting suitable methods for specific study populations [54]. Our results are in line with recent research which highlights significant differences resulting from the use of diverse cut-point methods and measurement approaches in preschool children and toddlers [25–28].

### Strengths and limitations

One of our study’s strengths is the successful collection of PA measurements in a large population of toddlers. Validated cut-point methods for the study population’s age were selected, and the epoch lengths and axes were adjusted to align with the calibration study settings. Furthermore, identical data processing was performed before the application of the cut-point sets. A limitation of the study is the exclusion of water-based activities from the measurement, which may have resulted in underestimating TPA, particularly during the summer months and in toddlers from the study site Tarragona, a seaside location. Additionally, other factors such as motor skills, sleep quality, health status, and environmental conditions may also impact PA and should be considered in future research. The protocol’s requirement for device removal during sleep and water-based activities created a significant dependence on parental compliance for proper device placement and device reapplication, which may have introduced methodological inconsistencies in data collection. To date, no non-wear algorithm has been specifically validated for use in toddlers, thus we used the Choi algorithm which was validated in adults. Furthermore, no observational data or criterion measures were available to verify our results and conclude which cut-point method is the best fit for our study population. A general limitation of PA and SB studies is the potential for reactivity bias, where wearing a device may lead parents to place greater emphasis on PA, thereby encouraging their children to be more active than usual [55].

## Conclusion

The Choice of cut-point methods has a substantial impact on the estimated time toddlers spend engaging in various intensities of PA based on accelerometer measurements. Notably, the use of a different cut-point method could lead to an about 10% difference in time spent in LPA and SB, but only a 1% difference for MVPA. Thus, results from studies that apply different data processing criteria are hardly comparable, and conclusions drawn from studies may suffer from inaccuracies. Furthermore, the choice of cut-points used can change the estimated adherence to established PA guidelines and thus the interpretation of study results. Thus, studies that associate PA with health outcomes may come to different conclusions depending on cut-point method choice. In toddlers, there is currently no consensus on the choice of cut-point methods. These findings provide essential evidence for countries implementing early childhood PA surveillance programs using accelerometery, highlighting the need for careful data interpretation and the development of validated standards to ensure comparability.

Future research is needed to standardize data analysis for a better comparability between studies analysing PA in toddlers, and to explore the relation between chosen cut-point methods and other measures of PA, or of health endpoints. As the movement capacities advance quickly with age in young children, it might be advantageous to define adequate cut-point methods for smaller age ranges. Additionally, waterproof devices that continuously monitor PA, SB, and sleep over a 24-hour period should provide even more accurate data because the data quality is not impacted by not captured PA or the need to identify non-wear periods. In future research, use of cut-point free methods or machine learning models could improve the classification of PA and SB in toddlers. 

## Supplementary Information

Supplementary Material 1

## Data Availability

The datasets analysed during the current study are available from the corresponding author on reasonable request.

## Abbreviations

BMI: Body mass index
CARS: Children´s Activity Rating Scale
CI: Confidence interval
ENMO: Euclidian norm of raw accelerations – 1 g
LPA: light physical activity
MPA: moderate physical activity
MVPA: moderate to vigorous physical activity
PA: physical activity
SB: sedentary behaviour
SD: standard deviation
TOMI: Toddler Milk Intervention
TPA: total physical activity
VA: vertical axis
VM: vector magnitude
VPA: vigorous physical activity
WHO: World Health Organization
