# Supplementary material for: Impact of cut-point methods on classification of physical activity and sedentary behaviour of toddlers
Source: BMC Public Health. 2025 Oct 2;25:3290. doi: 10.1186/s12889-025-24636-6 (PMC12492789; doi:10.1186/s12889-025-24636-6)
Supplement: Supplementary file 1 — Supplementary Material 1 [file 12889_2025_24636_MOESM1_ESM.docx]

Supplementary material

1. Weartime protocol

| Subject Name | On Date | On Time | Off Date | Off Time | Category^1^ |
| --- | --- | --- | --- | --- | --- |
|  |  |  |  |  |  |
|  |  |  |  |  |  |
|  |  |  |  |  |  |
|  |  |  |  |  |  |
|  |  |  |  |  |  |

^1^Category: includes a reason for removing the Actigraph, e.g. bathing or napping
